# Use of a Smartphone Platform to Help With Emergency Management of Acute Ischemic Stroke: Observational Study

**DOI:** 10.2196/25488

**Published:** 2021-02-09

**Authors:** Yiqun Wu, Fei Chen, Haiqing Song, Wuwei Feng, Jinping Sun, Ruisen Liu, Dongmei Li, Ying Liu

**Affiliations:** 1 Department of Epidemiology and Biostatistics School of Public Health Peking University Health Science Center Peking China; 2 Department of Neurology Xuanwu Hospital Capital Medical University Beijing China; 3 Department of Neurology Duke University School of Medicine Durham China; 4 Department of Neurology The Affiliated Hospital of Qingdao University Qingdao China; 5 Beijing Municipal Health Commission Beijing China; 6 BEIJING ANMED Medical Technology Co Ltd Beijing China

**Keywords:** acute ischemic stroke, door-to-needle time, smartphone platform, emergency management, smartphone, mHealth, stroke, management, emergency, first aid, utility, digital health

## Abstract

**Background:**

To improve the outcomes of acute ischemic stroke (AIS), timely thrombolytic therapy is crucial. Series strategies were recommended to reduce door-to-needle (DTN) time for AIS. Mobile technologies are feasible and have been used in stroke management for various purposes. However, the use of smartphone platforms that integrate series strategies through the entire first aid process to improve emergency management of AIS remains to be verified.

**Objective:**

This study aims to describe the utility and application of a smartphone platform in the emergency management of AIS and report the DTN time for patients with AIS during its 2-year application period. Our results are relevant to digital health management.

**Methods:**

A smartphone platform named “Green” was developed to incorporate the field assessment, hospital recommendation, prehospital notification, real-time communication, clinical records creation, key time-stamping, and quality control to streamline and standardize overall AIS emergency management processes. The emergency medical system (EMS) and all the emergency departments in Beijing have used this platform since 2018. From January 1, 2018, to December 31, 2019, 8457 patients diagnosed with AIS received intravenous tissue-type plasminogen activator therapy. The median DTN time and the proportions of patients with DTN times of ≤60 minutes and ≤45 minutes were reported.

**Results:**

During the 2-year application period of this platform, the median DTN time was 45 minutes, and the proportions of patients with DTN times of ≤60 minutes and ≤45 minutes were 74.6% and 50.5%, respectively. The median DTN time was significantly reduced from 50 minutes in 2018 to 42 minutes in 2019 (*P*<.001). The proportions of patients with DTN times of ≤60 minutes and ≤45 minutes increased from 66.1% and 40.7%, respectively, in 2018 to 80.7% and 57.3%, respectively, in 2019 (both *P*<.001). Sustained improvement in DTN time was seen during all the observed months. The improvement occurred across all facilities, and the variations among hospitals also decreased. The median DTN time for patients transferred by ambulances (43 minutes) was significantly shorter than those who reached hospitals by themselves (47 minutes; *P*<.001).

**Conclusions:**

Sustained reductions in DTN time reflected the improvement in AIS emergency management processes. The use of a smartphone platform integrating recommended strategies throughout all first aid stages is a practical way to help the emergency management of AIS.

## Introduction

Stroke is the second-leading cause of death and disability worldwide [1] and accounts for almost 10% of all deaths [2]. The number of people who remain disabled from stroke has almost doubled during the last 30 years. Ischemic stroke comprises 65% of all strokes [1]. To improve the outcomes of acute ischemic stroke (AIS), timely thrombolytic therapy is crucial [3,4]. For every 15-minute reduction in door-to-needle (DTN) time, there is an associated benefit of a 5.0% reduction in mortality [3]. Due to the importance of rapid treatment, guidelines recommend that DTN time should be capped at 60 minutes for patients with AIS [5]. According to published data, adopting 10 best-practice strategies could reduce DTN time by 15 minutes [4], and implementing an expanded 16 strategies could save 20 minutes [6]. Of these strategies, several can be carried out in the prehospital emergency medical services (EMS) system. Recent guidelines have advised prehospital EMS systems to be integrated into the early management of patients with AIS [7,8].

In the prehospital stage, integrating mobile technologies such as prehospital assessment and prehospital notification for AIS into the EMS system has been reported to be feasible and beneficial [9-12]. For example, the Field Assessment Stroke Triage for Emergency Destination (FAST-ED) app improves the triage of patients with AIS, reduces hospital arrivals times, and maximizes the use of thrombolytic therapy [9]. Mobile technologies have been widely used to improve the management of stroke in different stages for various purposes [13,14]. Nevertheless, reports on the use of smartphone platforms incorporated into the overall emergency management process—from the prehospital stage to subsequent admission for further in-hospital treatment—are limited [15]. We hypothesize that such smartphone platforms could improve prehospital and in-hospital coordination, facilitate and standardize the workflow, and improve emergency management.

In China, stroke has been the leading cause of death, with an increase of 46.8% in disability-adjusted life years (DALYs) in the last 30 years [16]. Like other countries, ischemic stroke (IS) was the major pathological type [17]. To improve the timely therapy for AIS, a series of policies and procedures have been implemented to set up the prehospital emergency care system, establish stroke care units in the emergency department (ED), and enhance coordination between prehospital care and ED in the stroke center [18]. The prehospital EMS was also recommended in the recent Chinese AIS therapy guideline [19]. To standardize and streamline the processes in prehospital and ED stroke care and strengthen prehospital and in-hospital coordination, a smartphone platform named “Green” was developed [20]. Herein, we describe the utility and application of Green in the emergency management of AIS and report the DTN times for patients with AIS during its 2-year application period.

## Methods

### Workflow for AIS Emergency Management With the Use of Green

#### Streamlining AIS Emergency Management Processes

**Figure 1:**
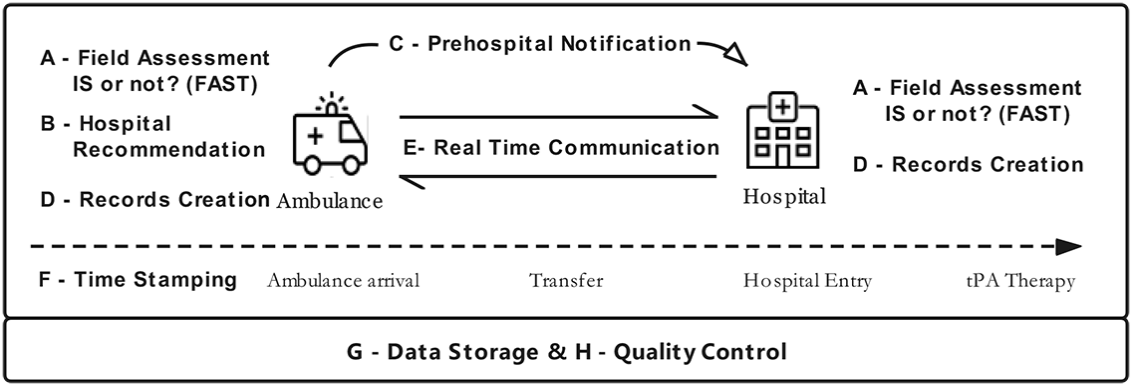
Application of the Green App in the workflow for acute ischemic stroke (AIS) prehospital and in-hospital treatment. FAST: Field Assessment Stroke Triage; H: hospital; IS: ischemic stroke; tPA: tissue-type plasminogen activator.

Green is a novel medical smartphone app developed jointly by the Beijing Municipal Health Commission and BEIJING ANMED Medical Technology Co Ltd in 2017. From January 2018, it has been freely available to the EMS systems and all eligible hospitals for AIS therapy in Beijing. Paramedics, physicians, and nurses in all stroke first aid facilities, as well as hospital management and quality control groups in Beijing, were trained several times to use this platform. In all, 8 aspects were integrated with the use of this app (Figure 1): (1) field assessment, (2) qualified hospital recommendation, (3) prehospital notification, (4) clinical records creation, (5) real-time communication, (6) time-stamping of events, (7) data storage, and (8) quality control.

#### Field Assessment for Stroke

Once the ambulance receives an emergency call and reaches the patient, paramedics will first assess whether it is a suspected IS according to 3 simple tests: face (does the face droop on one side when the person tries to smile?), arms (is one arm lower when the person tries to raise both arms?), and speech (can the person repeat a simple sentence without slurring?). If any of these signs are observed, paramedics will classify the patient as having a suspected IS. Then, paramedics will input the patient's demographic characteristics and activate the platform. If the patient reaches the hospital by themselves, doctors who receive the suspected IS patient will activate the platform and create relevant clinic records.

#### Qualified Hospital Recommendation

Once paramedics activate the platform with the demographic information of a suspected patient, the nearest qualified stroke center or hospital will show up on the screen according to the First Aid Treatment Map for Stroke (FATMS) published by Beijing Health and Family Planning Commission. Based on the real-time traffic information, the app can calculate the estimated arrival times from the patient's location to the nearest qualified hospitals on the FATMS. The paramedics will then choose either the nearest qualified hospital or one of the other nearby hospitals according to the patient's preference (if any) and send the patient's information to the hospital while transporting the patient there.

#### Prehospital Notification to the Receiving Hospital

At the same time, the ED in the selected hospital will be alerted to any new potential stroke case. If the chosen hospital has a spare treatment bed at the time, the ED stroke care team in the hospital will confirm the information, alert the entire therapy team, prepare computed tomography (CT) scans and other resources, and clear a fast pathway for the incoming patient. Meanwhile, paramedics in the ambulance will receive confirmation from the target hospital via the Green app and transfer the patient there accordingly. During the transportation, automated time-stamping of events can be seen from the app for both paramedics in the ambulance and the ED care team in the receiving hospital, as the app incorporates GPS information.

#### Clinical Records Creation

During transportation, paramedics will initiate the patient's clinical records, including the main complaint, disease onset time, vital signs, blood glucose levels, and electrocardiogram. Prehospital staff will obtain informed consent from patients' relatives for thrombolytic therapy. The ED care team in the receiving hospital has simultaneous access to the clinic records, which are updated in real-time. If the patients reach the hospital by themselves, doctors in the hospital create and complete clinic records for patients in the app. The records are then automatically entered into the electronic clinic records in the chosen hospitals.

#### Real-Time Communication Between Prehospital and In-Hospital

Real-time communication between medical care professionals in the ambulance and the ED in the receiving hospital can take place during the patient's transportation. Paramedics can conduct several prehospital therapies or prepare under the ED care team's instruction, such as initial emergency care and prehospital informed consent for possible thrombolytic therapy. When each link in the ED care team is in a position to receive the patient, their readiness status is updated through the real-time communication window to facilitate care coordination.

#### Semi-automated Time-Stamping of Events

Once paramedics activate the platform, the time is automatically recorded. Based on the input disease onset time, the interval time from then on will be automatically shown at the top of the screen until the thrombolytic therapy is completed. During the whole treatment, time records for several key points need to be inputted into the app, including times for images, informed consent, intravenous thrombolysis, endovascular treatment, and revascularization. The stroke care team can easily access the interval time from onset from the app.

#### Data Storage

The data collection terminal is accessed via HTTPS to implement channel encryption and other measures to ensure terminal communication security. The overall software system information security protection is in compliance with the Regulation of the People's Republic of China on Protecting the Safety of Computer Information Systems, which referred to the Grade III information system. Several security strategies were incorporated into the data storage system, including the protection strategy of partition Fenwick, security access, dynamic perception, lean management, and comprehensive protection. The all-around system protection covers physical, boundary, server, terminal, application, and data protection, including integrity protection, confidentiality protection, and backup and recovery protection, to keep the system data in a safe state without danger and infringement.

#### Quality Control

Using the time records for the key therapy points, outside quality control parties can compare and analyze the data, rate the IS first aid performance of each hospital and ED, adjust the qualification of IS first aid facilities, and renew the regional FATMS. Quality control groups inside hospitals can use the information to find weak points during the therapy courses, provide suggestions, and improve the AIS first aid performance thereafter. During the 2-year usage of this app, the Beijing Municipal Health Commission and other quality control parties released quality control reports every month to all the stroke first aid facilities, held stroke emergency therapy seminars across hospitals, and helped hospitals improve their ability to administer first aid.

### DTN Times for Patients With AIS

All the stroke first aid facilities in Beijing have used the application for 2 years since January 1, 2018. From the records in the platform, the DTN times of patients with AIS were analyzed in this report. DTN time was defined as the time interval between hospital arrival to initiation of intravenous tissue-type plasminogen activator (tPA) therapy. From January 1, 2018, to December 31, 2019, a total of 13,434 patients were diagnosed with AIS in Beijing, and of these, 13,058 (97.2%) patients had completed time records for key points during the acute treatment process. Among these 13,058 patients, 8457 (64.8%) received tPA, of whom 3186 (37.7%) arrived at the ED by ambulance (group A) and 5271 (62.3%) arrived by other transportation methods arranged by the family (group B). For group A, emergency management started from the time at which the paramedics reached the patients, whereas for group B, management started from the time of the patients' arrival to the hospital. For group A, all the functions in the Green app (stages 1-8) were involved in the management processes. For group B, several functions related to the EMS system, such as field assessment (stage 1), hospital recommendation (stage 2), prehospital notification (stage 3), and real-time communication (stage 5), were not applied; however, functions such as clinical records creation (stage 4), time-stamping of events (stage 7), and quality control (stage 8) were still applicable. The comparison between DTN times in the 2 groups would likely reflect the effect of management in the prehospital stage and the coordination between the prehospital and in-hospital processes.

### Statistical Analyses

The median DTN time and proportions of patients with DTN times of ≤60 minutes and ≤45 minutes were reported for the entire 2-year period, for each year and each month, respectively. The median DTN time for each hospital was calculated, and the standard deviation was used to represent the variation between hospitals. All results were calculated separately for groups A and B. The Mann-Whitney U-test and chi-square test were used to compare differences between the groups. *P* values less than .05 were considered to indicate statistically significant differences. All analyses were conducted using R (version 3.6.0; R Core Team) [21].

### Availability of Data and Materials

The statistical code is available upon reasonable request to the corresponding author.

## Results

**Figure 2:**
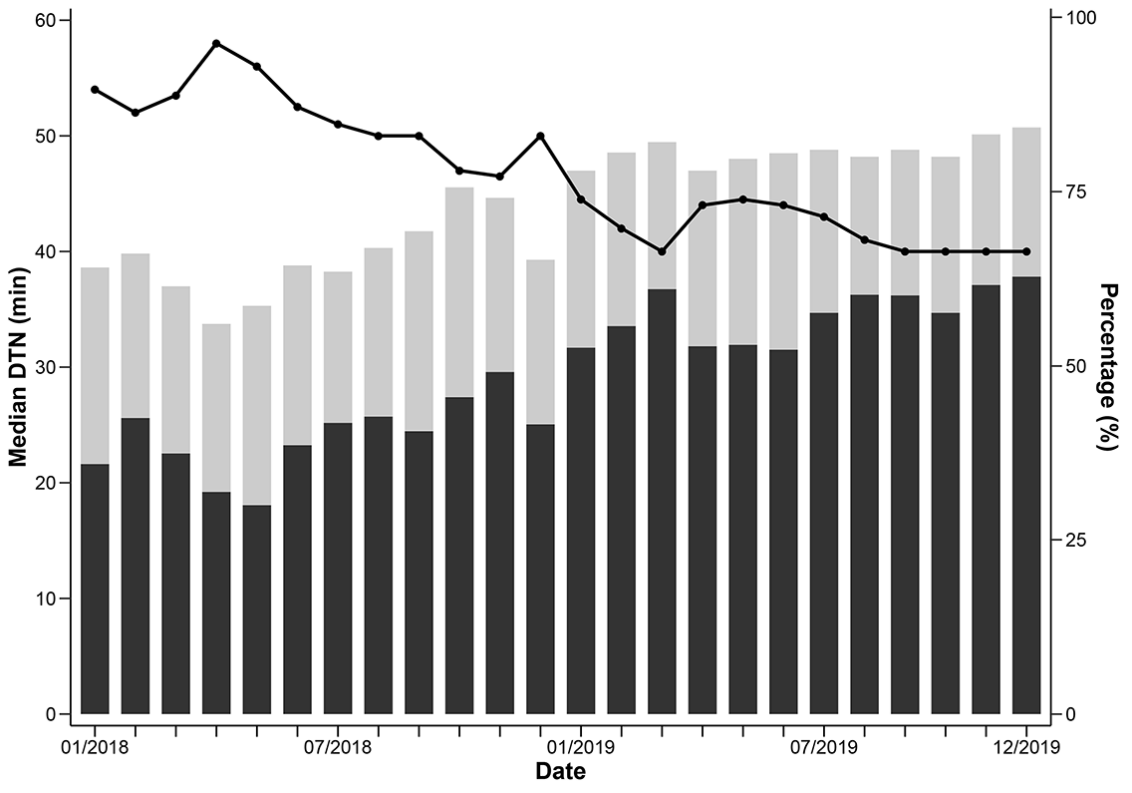
Door-to-needle (DTN) times for patients with acute Achemic stroke (AIS) who received intravenous tissue-type plasminogen activator (tPA) therapy from January 2018 to December 2019. Black bars: proportion of patients with DTN ≤45 minutes; dots and lines: median DTN times; grey bars: proportion of patients with DTN times ≤60 minutes.

**Figure 3:**
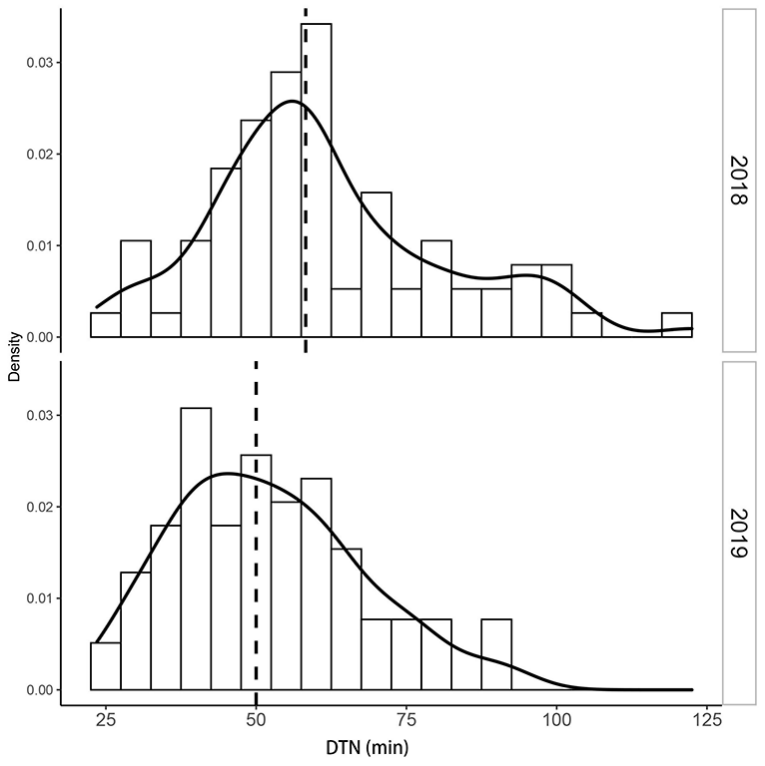
Distribution of door-to-needle (DTN) times among acute ischemic stroke (AIS) first-aid hospitals in Beijing.

From 2018 to 2019, the median DTN time for patients with AIS who accepted intravenous tPA therapy was 45 minutes (Table 1). The median DTN time in 2019 (42 minutes) was significantly shorter than that in 2018 (50 minutes; *P*<.001; Table 2). As seen in Figure 2, the median DTN time in each month decreased continuously during the 2 years, from 54 minutes in January 2018 to 40 minutes in December 2019 (*P*<.001). The improvement in DTN time was noted in all the IS first aid facilities; the median DTN time in each hospital shifted from the right to the left by more than 8 minutes, and the variation of median DTN times among hospitals also decreased from 19.9 minutes in 2018 to 15.5 minutes in 2019 (Figure 3).

**Table 1:** Door-to-needle (DTN) times for patients with acute ischemic stroke (AIS) transferred by ambulances vs. those that reached the hospitals by themselves.

DTN times (in minutes)	Overall (n=8457)	Group A^a^ (n=3186)	Group B^b^ (n=5271)	*P* value
Median (IQR)	45 (29)	43 (30)	47 (28)	<.001
≤60 minutes, n (%)	6314 (74.6)	2457 (77.1)	3857 (73.1)	<.001
≤45 minutes, n (%)	4267 (50.5)	1747 (54.8)	2522 (47.8)	<.001

^a^Group A: AIS patients transferred by ambulances.

^b^Group B: AIS patients who traveled to the hospitals by themselves.

**Table 2:** Door-to-needle (DTN) times for patients with acute ischemic stroke (AIS) who received intravenous tissue-type plasminogen activator (tPA) therapy in Beijing in 2018 and 2019.

DTN times (in minutes)		Overall	Group A^a^	Group B^b^	*P* value
**In 2018**					
	Sample Size, N	3504	1202	2302	
	DTN, median (IQR)	50 (34)	48 (35)	53 (34)	<.001
	DTN ≤ 60 minutes, n (%)	2316 (66.1)	846 (70.4)	1470 (63.9)	<.001
	DTN ≤ 45 minutes, n (%)	1427 (40.7)	564 (46.9)	863 (37.5)	<.001
**In 2019**					
	Sample Size, N	4953	1984	2969	
	DTN, median (IQR)	42 (27)	40 (27)	43 (26)	<.001
	DTN ≤ 60 minutes, n (%)	3998 (80.7)	1611 (81.2)	2387 (80.4)	0.51
	DTN ≤ 45 minutes, n (%)	2840 (57.3)	1181 (59.5)	1659 (55.9)	0.01

^a^Group A: patients with acute ischemic stroke who were transferred by ambulances.

^b^Group B: patients with acute ischemic stroke who reached hospitals by themselves.

Overall, among 8457 patients with AIS traveling to hospitals, 6314 (74.6%) and 4267 (50.5%) patients received intravenous tPA therapy within 60 minutes and 45 minutes, respectively (Table 1). The proportion of patients with DTN times that were ≤60 minutes and ≤45 minutes were 66.1% (2316/3504) and 40.7% (1427/3504) in 2018, respectively, while both proportions increased significantly (both *P*<.001) in 2019 to 80.7% (3998/4953) and 57.3% (2840/4953), respectively (Table 2). Continuous increases in these 2 proportions (DTN ≤60 minutes and ≤45 minutes) for each month was seen during the 2 years, with 64.1% and 35.9% in January 2018 and 84.2% and 62.8% in December 2019 (Figure 2).

The median DTN time for patients who were transferred by ambulance (43 minutes) was significantly shorter than those who reached the hospital by themselves (47 minutes; *P*<.001; Table 1). Accordingly, the proportion of patients both with DTN times of ≤60 minutes and ≤45 minutes were significantly higher in patients transferred by ambulance (both *P*<.001; Table 1). Similar results were seen in both 2018 and 2019 (Table 2). For both groups, the DTN times and proportions with DTN times of ≤60 minutes and ≤45 minutes each month showed continuous improvement (Multimedia Appendix 1).

## Discussion

### Principal Results

In this study, we described a smartphone freeware app developed for the emergency management of AIS through the entire first aid process. In the 2 years of use for standardizing the emergency management of AIS, the DTN time decreased significantly, with substantial increases in the proportion of patients with DTN times of ≤60 minutes and ≤45 minutes. Several strategies incorporated in the process accounted for the improvement, including hospital prenotification by EMS, rapid triage protocol, enhanced coordination, communication among providers inside and outside the hospital, key point time trackers, and prompt patient-specific data feedback to the EMS providers and stroke team. These strategies were among the 10 best stroke therapy practice strategies recommended by the American Heart Association/American Stroke Association (AHA/ASA) [22]. According to the results from 71,169 stroke patients, the DTN time decreased by about 15 minutes after implementing the 10 best strategies [4]. Moreover, the DTN time may decrease by about 20 minutes if 16 strategies recommended by AHA/ASA were implemented together [6].

### Comparison With Prior Work

Using electronic apps is a feasible way to implement multiple recommended strategies. Reportedly, the Stop Stroke app, which can enhance coordination and communication between the inside and outside of hospitals, improved DTN time by a decrease of 21 minutes [23]. The reasons for the improvement were multifactorial [15]. Similarly, the Green app's use in this study improved DTN time significantly by implementing several management processes. Moreover, the improvement was continuous from the first months after its application to the 24 months thereafter. According to previous studies, for every 15-minute reduction in DTN time, an associated improved mortality benefit of 5.0% could be obtained [3]. Therefore, significantly improved mortality benefits may be obtained as a result of the continuous improvement in DTN time.

According to our results, an improvement in DTN was seen in all the first aid facilities, along with a significant decrease in the variations among facilities. The variations among facilities were influenced by multiple factors [24]. It was reported that hospital variation accounted for 12.7% of the variability in DTN times [25]. Since January 2018, all the first aid facilities in Beijing began to use this application. The standardized management processes among facilities after applying a unique platform was the major reason for the reductions in the interhospital variations. Moreover, the prompt patient-specific data feedback may have also contributed to the changes. The data feedback system can help different stroke teams identify their specific delays and devise strategies to overcome these barriers [6]. Due to a systematic improvement in all the facilities, further health benefits would be obtained, along with an improvement in the equality for AIS treatment in the whole region.

According to the results, patients who were transferred by ambulances had shorter DTN times than those who went to hospitals by themselves. This result was similar to those reported in previous studies [24]. Prehospital EMS systems and advanced hospital notifications may play significant roles in the differences between both groups. The EMS triage and advanced hospital notification by EMS has been reported to significantly reduce DTN time [6,26]. Moreover, in the standard management processes in our study, paramedics would explain the possibility of intravenous therapy, as well as the potential benefits and side effects of the therapy, to the patient's relatives during the transfer. If possible, pre-informed consent will be signed by relatives. This process can further save time for in-hospital consent. It has been reported that a waiver of written informed consent before tPA administration could save about 0.8 minutes [6]. From 2018 to 2019, there were only about 40% of patients with IS transferred by ambulances. In the future, an improvement in the usage of the prehospital EMS system would further improve the DTN time for patients with AIS.

### Limitations

Our study has some limitations. First, because of the lack of information before the study period and the lack of a parallel control group during the study period, we were unable to estimate the magnitude of DTN improvement owing to the processes we implemented. Despite this, continuous reductions in DTN time were observed during the study period, and the reductions happened in all hospitals. The improvement was a combined effect from fast EMS triage, prehospital notification, enhanced coordination, and a prompt feedback process. Further studies should explore to what extent each strategy can influence the outcome. Second, spatial factors may influence the usage and performance of the platform. In this study, the platform was developed and used in Beijing, an overpopulated city with a large number of acute stroke care centers. Compared with peripheric city areas, the performance of the platform may be different because of the current treatment workflow, capacity, and efficiency of the emergency medical system and emergency departments in the hospitals, as well as the traffic conditions and distribution of acute care centers in the city. When more data is available, the performances of the platform in peripheric city areas should be further evaluated. Third, the long-term health and economic benefits should be evaluated when data are available.

### Conclusions

A smartphone app was developed to streamline the emergency management of AIS in Beijing since 2018. Sustained reductions in DTN time were observed, reflecting the improvement in management processes. The use of a smartphone platform was a feasible way of integrating recommended strategies to help the emergency management of AIS. Similar measures are recommended in other areas with high AIS risks.

## Appendix

Multimedia Appendix 1 Door-to-needle (DTN) times for patients with acute ischemic stroke (AIS) transferred by ambulances or who reached hospitals by themselves, from January 2018 to December 2019. (A) DTN times for patients with AIS transferred by ambulances; (B): DTN times for patients with AIS who reached hospitals by themselves. Black bars: proportions of patients with DTN ≤ 45 minutes; dots and lines: median DTN times; grey bars: proportions of patients with DTN ≤ 60 minutes.

## Abbreviations

AHA/ASA: American Heart Association/American Stroke Association
AIS: acute ischemic stroke
DTN: door-to-needle
ED: emergency department
EMS: emergency medical system
FATMS: First Aid Treatment Map for Stroke
IS: ischemic stroke
tPA: tissue-type plasminogen activator
