# Supplementary material for: Use of a Smartphone Platform to Help With Emergency Management of Acute Ischemic Stroke: Observational Study
Source: JMIR Mhealth Uhealth. 2021 Feb 9;9(2):e25488. doi: 10.2196/25488 (PMC7902188; doi:10.2196/25488)

**Multimedia Appendix 1**. Door-to-needle (DTN) times for patients with acute ischemic stroke (AIS) transferred by ambulances or who reached hospitals by themselves, from January 2018 to December 2019. (A) DTN times for patients with AIS transferred by ambulances; (B): DTN times for patients with AIS who reached hospitals by themselves. Black bars: proportions of patients with DTN ≤ 45 minutes; dots and lines: median DTN times; grey bars: proportions of patients with DTN ≤ 60 minutes.


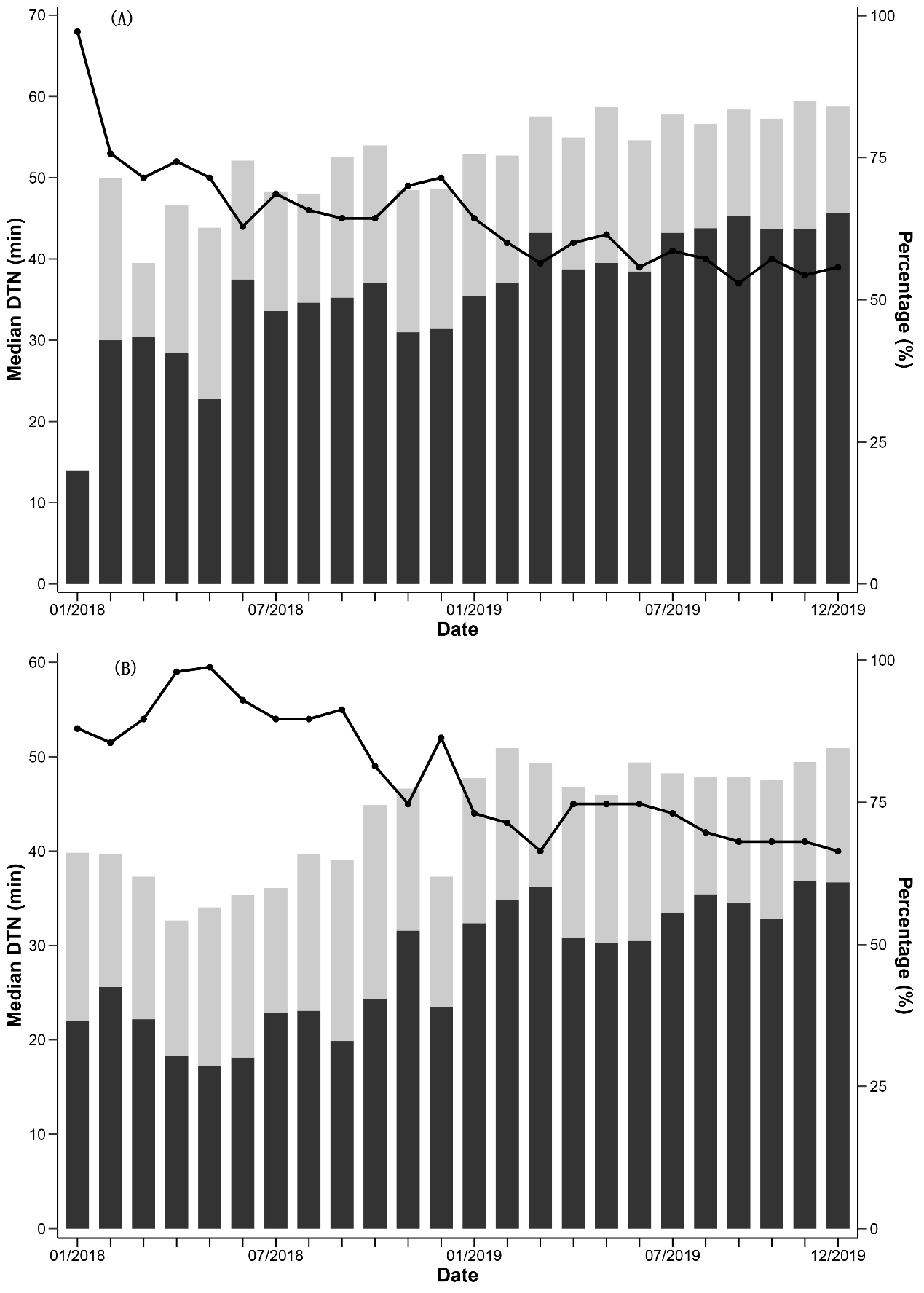

Supplement: Multimedia Appendix 1 [file mhealth_v9i2e25488_app1.docx]
